# Inclusion bodies as potential vehicles for recombinant protein delivery into epithelial cells

**DOI:** 10.1186/1475-2859-11-67

**Published:** 2012-05-24

**Authors:** Mirjana Liovic, Mateja Ozir, Apolonija Bedina Zavec, Spela Peternel, Radovan Komel, Tina Zupancic

**Affiliations:** 1 Medical Center for Molecular Biology, Faculty of Medicine, University of Ljubljana, Ljubljana, Slovenia; 2 National Institute of Chemistry, Hajdrihova 19, Ljubljana, Slovenia

**Keywords:** Inclusion bodies, E. coli, Keratin, Intermediate filaments, Skin, Therapy, Electroporation

## Abstract

**Background:**

We present the potential of inclusion bodies (IBs) as a protein delivery method for polymeric filamentous proteins. We used as cell factory a strain of *E. coli,* a conventional host organism, and keratin 14 (K14) as an example of a complex protein. Keratins build the intermediate filament cytoskeleton of all epithelial cells. In order to build filaments, monomeric K14 needs first to dimerize with its binding partner (keratin 5, K5), which is then followed by heterodimer assembly into filaments.

**Results:**

K14 IBs were electroporated into SW13 cells grown in culture together with a “reporter” plasmid containing EYFP labeled keratin 5 (K5) cDNA. As SW13 cells do not normally express keratins, and keratin filaments are built exclusively of keratin heterodimers (*i.e.* K5/K14), the short filamentous structures we obtained in this study can only be the result of: a) if both IBs and plasmid DNA are transfected simultaneously into the cell(s); b) once inside the cells, K14 protein is being released from IBs; c) released K14 is functional, able to form heterodimers with EYFP-K5.

**Conclusions:**

Soluble IBs may be also developed for complex cytoskeletal proteins and used as nanoparticles for their delivery into epithelial cells.

## Background

When expressing recombinant proteins in bacteria, one of the most common side effects is the formation of inclusion bodies (IBs) [1]. Until recently IBs were thought of as blocks of misfolded and inactive proteins. As it was difficult and expensive to purify and refold recombinant proteins from such aggregates, a whole range of strategies was adopted to reduce or avoid IB formation. Surprisingly, although there was published evidence that inclusion bodies can also contain biologically active proteins [2,3], it was not until recently that this phenomenon was fully acknowledged and further investigated [4,5]. Many different protocols for IB production have been published, focusing on the various parameters that can affect IB quality such as the genetic background of the host organism, specific growth conditions and IB isolation [6-18]. Furthermore, the term “non-classical” inclusion bodies (ncIBs) was introduced to describe IBs containing a significant amount of properly folded protein, which in addition could be extracted under non-denaturing conditions. Although the major factor in obtaining soluble IBs is the target protein’s structure, the type of host organism used and the design of the biosynthesis process may also modulate IB size and degree of solubility. The fact that inclusion bodies may contain biologically active proteins has been already shown for a number of proteins, from GFP [4,16,17], to recombinant cytokines such as granulocyte colony-stimulating factor [5] and a number of enzymes [19-23]. Therefore, the development of IBs as a novel type of recombinant protein delivery machines has great potential with applications in medicine, and it has been also recently patented [24].

The aim of this study was to provide evidence that IBs may be also used as method of recombinant protein delivery for complex proteins. By using an already published protocol for IB production *via* the *E. coli* BL21 (DE3) strain [17], we obtained keratin 14 (K14) ncIBs of several hundred nanometres in size, which were subsequently introduced into SW13 epithelial cells grown in culture by means of electroporation. We show that inside the cells a portion of the K14 protein is released and dimerizes with EYFP labeled K5, resulting in short filamentous structures, precursors of keratin filaments.

## Results and discussion

**Figure 1:**
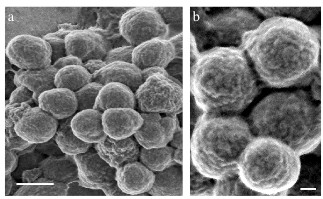
**Field emission scanning electron microscopy of K14 inclusion bodies.** (**a**) Most obtained K14 IBs were spherical in shape. Scale bar 500 nm. (**b**) A high power magnification of K14 IBs, scale bar 100 nm.

As shown in Figure 1, the majority of the K14 IBs we tested in this study were spherical in shape and approximately 400–500 nm in size. High power magnification (Figure 1B) proved that they consisted of much smaller nuclei of accumulated protein, in turn attached to each other forming a complex higher order structure.

**Figure 2:**
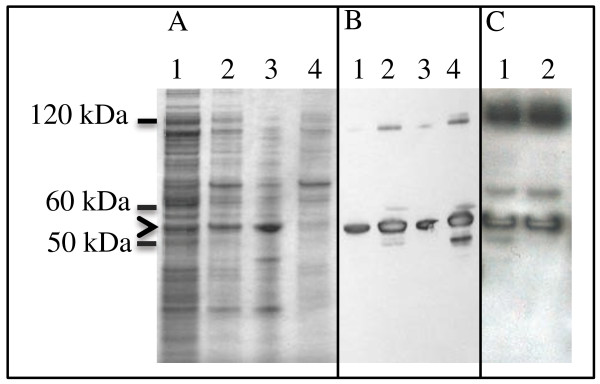
**SDS-PAGE gel analysis of K14 inclusion bodies.(A)** Comassie stained SDS-PAGE gel of proteins extracted from bacterial cell lysates: 1, soluble (cytoplasmic) proteins; 2, IB proteins; 3, IB proteins extracted with 0.2% NLS; 4, IB proteins insoluble in 0.2% NLS. **(B)** Western blot analysis of the same protein extracts as in A, however imunoblotted against K14: 1, soluble (cytoplasmic) proteins; 2, IB proteins; 3, IB proteins extracted with 0.2% NLS; 4, proteins insoluble in 0.2% NLS. **(C)** Western blot analysis of K14 IBs solubilized over night in 1, Epilife serum-free medium, and 2, DMEM medium. The arrow points out bands corresponding to K14, which has a molecular weight of 51 kDa.

The obtained K14 IBs displayed a significant level of solubility. Proteins extracted from bacterial cells were first resolved on a Comassie stained SDS-PAGE gel (Figure 2A). Lanes 3 and 4 (Figure 2A) correspond to the soluble and insoluble fractions of extracted K14 IBs, dissolved in a mild detergent solution (0.2% N-lauroylsarcosine). Figure 2B shows an immunoblot of the same bacterial cell extracts with an antibody raised against K14, confirming that the strong band visible after Comassie staining (approx. 51 kDa, marked with an arrow) is indeed K14. Furthermore, we demonstrate that our K14 IBs are partially soluble already in commercially available cell culture media, such as Epilife serum-free medium (Invitrogen) (Figure 2C, lane 1), and DMEM (Figure 2C, lane 2). As the obtained K14 IBs are soluble under non-denaturing conditions, they may be classified as non-classical IBs (ncIBs).

**Figure 3:**
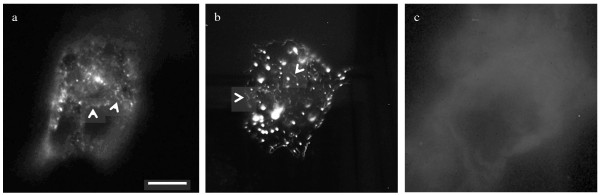
**Epi-fluorescent microscopy of electroporated SW13 cells.** (**a**) SW13 cells electroporated with a mixture of K14 ncIBs and a plasmid containing EYFP labeled K5 cDNA. (**b**) The positive control experiment, where cells were electroporated with plasmid DNAs encoding for EYFP-K5 and EYFP-K14. (C) The negative control experiment, where cells were electroporated exclusively with EYFP-K5 plasmid DNA. Experiments in (**a**) and (**b**) resulted in a multitude of speckles and short filamentous structures (see arrows). The scale bar is 20 μm.

To prove that our K14 ncIBs contain functional K14, we performed a series of electroporation experiments on SW13 cells (a human adrenal carcinoma cell line). As SW13 cells do not normally express keratins, and keratins are obligate heterodimers (build heterodimer subunits with a particular keratin partner), both K14 (in the form of ncIBs) and its binding partner, K5 (in the form of plasmid DNA containing EYFP labelled K5 cDNA), need to be simultaneously introduced into these cells. Short K5/K14 fragments (filament precursors) will form not only if the K14 protein is released out of the K14 ncIBs, but also if it is able to heterodimerize with overexpressed EYFP-K5 and these subunits are in turn able to stack up (polymerize) into filament fragments. In Figure 3 are presented the results of such *de novo* filament formation experiments, at the stage of 2 days after electroporation. Panel (A) is representative of SW13 cells electroporated with a mixture of K14 ncIBs and EYFP-K5 plasmid DNA. Panel (B) is representative for the positive control experiment, consisting of cells electroporated with plasmid DNAs encoding for EYFP-K5 and EYFP-K14. To demonstrate that overexpressed EYFP-K5 on its own does not form any filamentous structures and only gives rise to background staining, we also performed a negative control experiment (panel C). In this case SW13 cells were electroporated only with the EYFP-K5 plasmid, resulting in a “milky” fluorescent background. Therefore, the background visible in (A) is due to EYFP-K5 plasmid DNA overexpression and the presence of unpolymerized EYFP-K5 protein: the quantity of synthesized protein by plasmid DNA overexpression is much greater than the amount of soluble K14 protein introduced into cells by means of ncIBs. In panel (B), monomeric K5 and K14 proteins are produced in more equimolar amounts due to both EYFP-K5 and EYFP-K14 plasmid DNAs overexpression, and these are constantly being used up through K5/K14 dimerization. Under the presented experimental conditions and in a keratin-null cell line such as SW13, the rate-limiting factor for K5/K14 filament formation is the quantity of available functional K14. In the experiment in panel (A), K14 is delivered by means of K14 IBs, while in panel (B) by EYFP-K14 plasmid DNA overexpression.

Overall, the results of these two experiments (Figure 3A with B) are similar, as in both experiments dot-like particles, larger speckles and short filamentous structures (see arrows) are visible. Importantly, although the K14 ncIBs used in these experiments were not purified in any particular way after extraction from bacterial cells, no cytotoxic effect was observed even at longer time points (7 days) after electroporation (data not shown).

## Conclusions

We provide for the first time evidence that a polymeric cytoskeletal protein may be produced in the form of inclusion bodies and used in this form to supplement epithelial cells lacking it. The production is cost effective (cheap), no particular purification step is needed, as our ncIBs appear not to be cytotoxic, and with the end result similar to the effect of a plasmid DNA construct. In this study electroporation was chosen as method for K14 ncIB delivery because of their size (currently several hundred nanometers). During electroporation small hydrophilic pores are transiently formed in the cell membrane, and this method has been demonstrated to work well also in *in vivo* experiments [25-27], particularly on skin. Nevertheless, other approaches may work as well, especially in the case of delivery of smaller IBs, which is something that needs to be further investigated. A choice of a different *E. coli* strain as cell factory could help improve the biosynthesis process, however there have been also reports on the advantages of host organisms such as gram-positive bacteria (*Bacillus sp.*), yeast and fungi [28-30], which can circumvent some of the limitations of *E. coli*. In fact, the choice of a different host organism may be the next appropriate step towards the optimization of this particular ncIB synthesis protocol, as a significantly higher proportion of properly folded protein needs to be delivered in order to achieve a therapeutic effect. The data presented in this manuscript is extremely promising, as it indicates that soluble IBs may be also obtained for cellular proteins that exert their function by building intricate polymeric structures. As skin is the most accessible organ by far, this method may particularly find possible applications in dermatology and the cosmetics industry.

## Material and methods

### Cell culture, electroporation and microscopy

Human adrenal carcinoma cells (SW13 cell line) were grown in DMEM (PAA, Austria) supplemented with 10%FBS (PAA, Austria) and an antibiotic-antimicotic reagent (cat no 15240–062, Gibco), at 37°C and 5% CO_2_.

Cells were cultivated in T75 cm^2^ cell culture flasks until confluence. Before electroporation, cells were trypsinized and counted. In each electroporation experiment 5x10^6^ cells were used.

IBs were resuspended in PBS buffer and an aliquot was subsequently diluted in electroporation buffer. Approximately 150 μg of K14 IBs and 10 μg of plasmid DNA (with EYFP-K5) was mixed and electroporated into SW13 cells using a Gene Pulser (Bio-Rad) electroporator set at 125 μF capacitance extender, 200 Ω pulse controler, 25 μF gene pulser capacitance and 1.5 kV voltage. In the positive control experiment 10 μg of each plasmid DNA (EYFP-K5 and EYFP-K14) were mixed and electroporated under the following Gene-Pulser settings: capacitance extender 125 μF, pulse controler 200 Ω, gene pulser capacitance 3 μF, voltage 2.5 kV. After electroporation cells were plated out onto 13 mm coverslips and incubated for a further 24 hrs, 48 hrs, 72 hrs and 7 days. At each time point cells were fixed with methanol-acetone (1:1), immunostained with a primary mouse monoclonal antibody against K14 (LL001) followed by a goat-anti-mouse FITC labeled secondary antibody (Alexa Fluor, Molecular Probes). Coverslips were analyzed on a Zeiss T200 inverted microscope operated by Zeiss Axiovision software and representative images were taken with a Zeiss Axiocam colour camera.

### Keratin constructs and inclusion bodies production

Keratin 14 (KRT14) cDNA sequence was cloned into pET23b expression plasmid (Novagen) using the *Nde I* restriction site. A previously published protocol for IB production was used [17]. In brief, the *E. coli* BL21 (DE3) strain (Novagen) was used. Inclusion bodies were produced in a shake culture flask with GYSP/amp100 medium, at 25°C and 160 rpm, until the culture reached the stationary phase. At this point bacterial cells were disrupted using a high-pressure homogenizer Emulsiflex-C5 (Avestin) at operating pressure 75–100 MPa. After cell disruption IBs were washed twice with PBS buffer and stored for further analysis and applications. The supernatant (soluble protein fraction) was also stored for analysis.

Keratin 5 cDNA sequence was cloned into the pEYFP-C1 plasmid (Clontech) using *Eco RI* and *Bam HI* restriction sites.

### K14 protein extraction from IBs

IBs resuspended in solubilizing buffer (40 mM Tris/HCl with 0.2% N-lauroyl sarcosine, pH 8.0) were incubated in a shaker for 24 hrs at 20°C, then centrifuged at 4400 g for 15 minutes. N-lauroyl sarcosine was removed using a Dovex 1 x 4–50 ion exchange resin (Sigma).

In a parallel experiment IBs were also dissolved in DMEM after a 24 hrs incubation at 20°C in a shaker. The soluble and insoluble fractions were separated by centrifugation at 4400 g for 15 minutes. Samples were analyzed on 4-12% Nu-PAGE Bis-Tris SDS-PAGE gels (Invitrogen) and stained with Colloidal Blue (Invitrogen). Western blot detection was performed using a primary mouse monoclonal antibody against K14 (LL001) followed by a secondary HRP labeled anti-mouse secondary antibody (Sigma).

### Scanning electron microscopy

IBs were thoroughly washed in pure water, mounted on gold-coated polycarbonate membrane filter with a 0.22 μm pore size (Isopore^TM^, Millipore). Samples were analyzed using a high-resolution Zeiss SUPRA 35 VP field emission scanning electron microscope.

## Competing interests

The authors declare no competing interests.

## Authors’ contributions

M.L. conceived the experiments and wrote the manuscript. M.L., M.O., A.B.Z., S.P. and T.Z. designed the experiments. M.L. prepared the keratin expression constructs, performed epifluorescent microscopy and data analysis. S.P. prepared the inclusion bodies and performed protein gel analysis. M.O. performed cell culture and immunostaining. T.Z. performed cell culture. A.B.Z. performed electroporation experiments. R.K. was consolidating author and participated in the manuscript preparation. All authors read and approved the manuscript.
